# The Importance of the Tumor Microenvironment and Hypoxia in Delivering a Precision Medicine Approach to Veterinary Oncology

**DOI:** 10.3389/fvets.2020.598338

**Published:** 2020-11-12

**Authors:** Mark Gray, James Meehan, Arran K. Turnbull, Carlos Martínez-Pérez, Charlene Kay, Lisa Y. Pang, David J. Argyle

**Affiliations:** ^1^The Royal (Dick) School of Veterinary Studies and Roslin Institute, University of Edinburgh, Scotland, United Kingdom; ^2^Translational Oncology Research Group, Institute of Genetics and Molecular Medicine, Western General Hospital, University of Edinburgh, Scotland, United Kingdom; ^3^Breast Cancer Now Edinburgh Research Team, Institute of Genetics and Molecular Medicine, Western General Hospital, University of Edinburgh, Scotland, United Kingdom

**Keywords:** precision medicine, tumor microenviroenment, implantable technologies, genomics, one-health

## Abstract

Treating individual patients on the basis of specific factors, such as biomarkers, molecular signatures, phenotypes, environment, and lifestyle is what differentiates the precision medicine initiative from standard treatment regimens. Although precision medicine can be applied to almost any branch of medicine, it is perhaps most easily applied to the field of oncology. Cancer is a heterogeneous disease, meaning that even though patients may be histologically diagnosed with the same cancer type, their tumors may have different molecular characteristics, genetic mutations or tumor microenvironments that can influence prognosis or treatment response. In this review, we describe what methods are currently available to clinicians that allow them to monitor key tumor microenvironmental parameters in a way that could be used to achieve precision medicine for cancer patients. We further describe exciting novel research involving the use of implantable medical devices for precision medicine, including those developed for mapping tumor microenvironment parameters (e.g., O_2_, pH, and cancer biomarkers), delivering local drug treatments, assessing treatment responses, and monitoring for recurrence and metastasis. Although these research studies have predominantly focused on and were tailored to humans, the results and concepts are equally applicable to veterinary patients. While veterinary clinical studies that have adopted a precision medicine approach are still in their infancy, there have been some exciting success stories. These have included the development of a receptor tyrosine kinase inhibitor for canine mast cell tumors and the production of a PCR assay to monitor the chemotherapeutic response of canine high-grade B-cell lymphomas. Although precision medicine is an exciting area of research, it currently has failed to gain significant translation into human and veterinary healthcare practices. In order to begin to address this issue, there is increasing awareness that cross-disciplinary approaches involving human and veterinary clinicians, engineers and chemists may be needed to help advance precision medicine toward its full integration into human and veterinary clinical practices.

## Introduction

Precision or personalized medicine endeavors to enhance patient outcomes by treating individuals based on certain factors. These factors can include disease biomarkers and molecular signatures at the cellular level but also the phenotype, environment, and lifestyle of the individual (1). While precision and personalized medicine are similar concepts, there are some differences in their definitions. Precision medicine greatly depends on data collection, data analysis and information whereas personalized medicine is a healthcare model that takes into account patient genetics with consideration of patient preferences, beliefs, attitudes, and social background. However, as a result of concerns that the phrase “personalized” might be misinterpreted, possibly leading patients to believe that unique treatments and/or drugs were being developed particularly for themselves, “personalized medicine” has now largely been replaced with the term “precision medicine” (2, 3).

The National Research Council in America (4) adopted the definition of precision medicine from the President's Council of Advisors on Science and Technology in 2008 as: “The tailoring of medical treatment to the individual characteristics of each patient…to classify individuals into subpopulations that differ in their susceptibility to a particular disease or their response to a specific treatment. Preventative or therapeutic interventions can then be concentrated on those who will benefit, sparing expense and side effects for those who will not.” As the definition implies, the overall aim of precision medicine is to provide the most effective treatment for a patient (5), enhancing the quality of care whilst also decreasing the use of unnecessary diagnostic tests and therapies, thereby reducing costs and side effects (6).

### The Precision Medicine Initiative

Precision medicine has obtained increased awareness in recent years within both human and veterinary research and clinical communities. The announcement of the “Precision Medicine Initiative” by Barack Obama during the State of the Union Address in 2015 led to increased media coverage and awareness within the lay community. This initiative sought to encourage research in the field of precision medicine in an effort “to bring us closer to curing diseases like cancer and diabetes—and to give us all access to the personalized information we need to keep ourselves and our families healthier” (7). The Precision Medicine Initiative, now termed “All of US,” plans to register over 1 million participants (8). Those enrolled are expected to disclose any data produced from sequencing, digital healthcare technologies and electronic medical records over a 10 year period. These data will then be examined to increase our understanding of disease biology and pathogenesis whilst providing data to enable a precision-led healthcare approach for both individuals and the population as a whole.

The former Prime Minister of the UK, David Cameron, launched the 100,000 Genomes Project in 2012 (9). This project was led by Genomics England and the National Health Service and aimed to use whole-genome sequencing to improve the management of patients diagnosed with cancer and rare inherited diseases. 13 Genomic Medicine Centers were created to carry out this programme and in 2018 this initiative reached its goal of sequencing 100,000 whole genomes. This project not only provided whole-genome sequencing data for patient treatment selection or enrolment into clinical trials, but also enabled researchers to have access to anonymous clinical and genomic data sets. In veterinary medicine in the UK, “Dogslife” was the first national longitudinal canine health program to be set up. Launched in 2010, the study aims to identify genetic and environmental risk factors for canine diseases and use the information to generate disease risk reduction approaches (10).

The feasibility of implementing the precision medicine initiative has improved in recent years largely due to decreased costs associated with high-throughput DNA sequencing, the implementation of electronic medical records across the country and the utilization of advanced imaging systems that have the ability to assess the tumor microenvironment (TME). Additional genome-based technologies are also progressively being employed as either diagnostic assays to categorize disease, or as prognostic or predictive tests. Collectively, these approaches have been seen as the foundation for a new molecular disease classification system which will deliver a more accurate means by which clinicians can screen for and discover disease at its earliest stages. This ultimately will enable the selection of drugs and/or treatments directed by individual patient genetics. Since disease evolution from baseline risk to clinical symptoms frequently takes many years, intermittent molecular and digital profiling is expected to advance healthcare approaches from acute intervention and disease control toward a greater emphasis on pro-active management of disease risks and eventually disease prevention (8).

**Figure 1:**
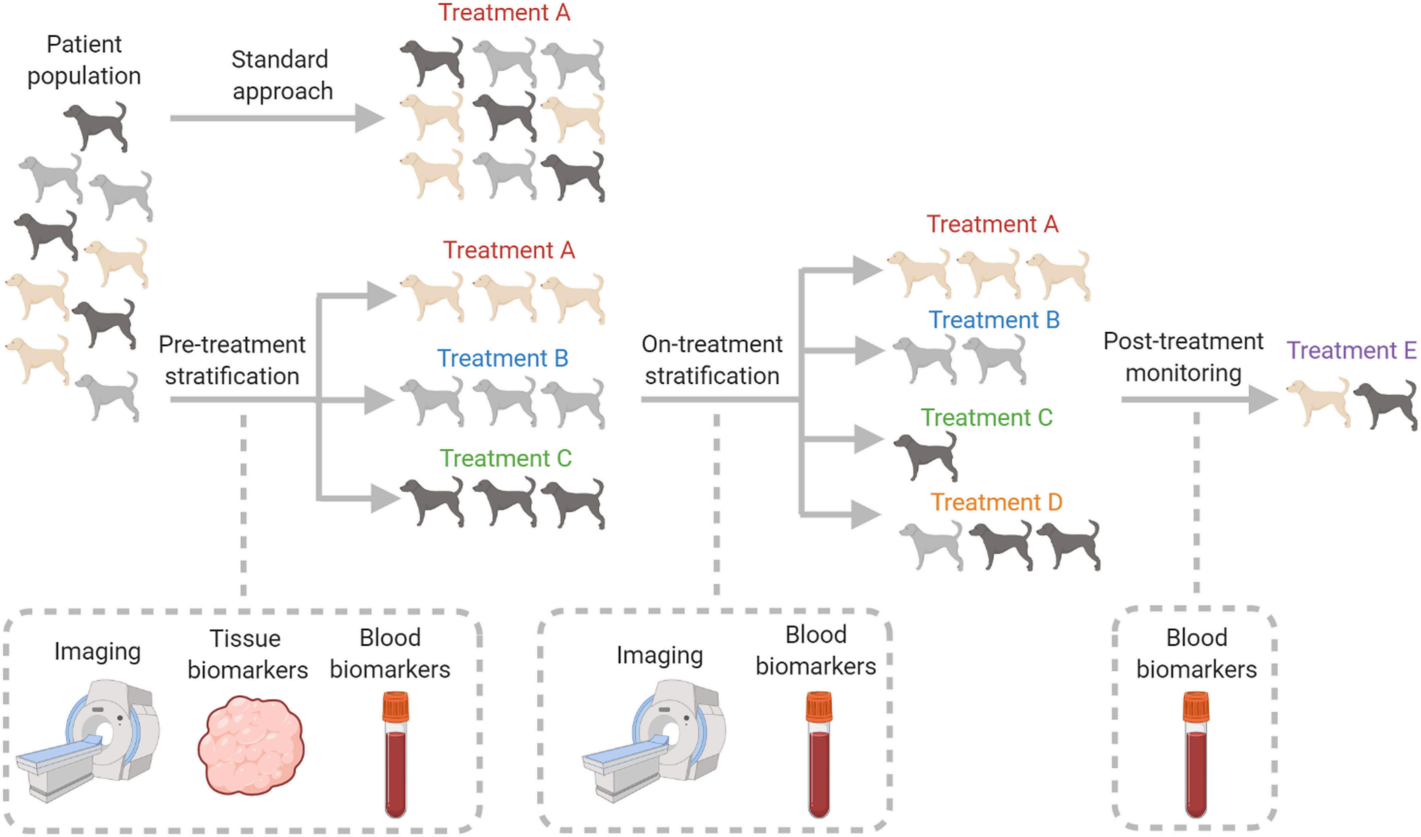
Comparison of the currently employed traditional healthcare practices with prospective precision medicine approaches in veterinary oncology. Veterinary patients presenting with the same tumor type are currently classified by their clinical stage and histological grade. This usually leads to a dichotomic decision to treat with standard specific treatment protocols, or not to treat at all. The vision of precision medicine is to develop diagnostic and monitoring techniques, applied to tumors of the same histological grade and clinical stage, to distinguish an optimized treatment strategy for each individual patient. Figure created with BioRender.com.

Oncology is perhaps one field that precision medicine can be most readily applied to. Cancer is a heterogeneous disease and although patients may be diagnosed with the same histological cancer type, their tumors may have varying genetic mutations or TMEs that lead to different responses between patients treated in the same way (11). Distinct treatment approaches for individual patients may therefore lead to improved outcomes (12). Within the field of oncology there is increasing awareness that cross-disciplinary approaches involving human and veterinary clinicians, engineers, and chemists may be needed to make significant progress in the field of precision medicine. In this review we discuss precision medicine with particular emphasis on the TME and describe how multidisciplinary groups are investigating the use of novel implantable technologies to achieve precision medicine. Finally, we describe a range of veterinary clinical studies that have used a precision medicine approach in a range of cancers in an attempt to improve patient outcome (Figure 1).

### The Tumor Microenvironment

Assessing the TME to improve patient treatment can be considered an important aspect of precision medicine. Stephen Paget initially put forward the concept of the TME in his seed and soil theory, which postulated that cancer development and progression are regulated by the interaction of cancer cells with the external environment of the tumor (13). The TME is a complicated network, comprising of not only the tumor cells themselves but also various tumor-associated cells. These associated cells include cancer stem cells, cancer-associated fibroblasts, mesenchymal stem cells, pluripotent stromal cells, cancer-associated adipocytes, endothelial cells, pericytes, and tumor-associated immune and inflammatory cells. These tumor-associated cells secrete soluble molecules and microvesicles that control the interactions of cancer cells with other cell types. This can influence how the tumor cells proliferate, oppose apoptosis, avoid elimination from the immune system, preserve stemness, invade, and metastasize (14).

The physiology of cancers differs greatly from that of normal tissues within the body; this situation results from the tumor needing to develop its own blood supply as it begins to outgrow the accessible vasculature from the organ in which it originates. However, the tumor neovasculature is crude and disordered, suffering from many functional and structural irregularities. This tumor vasculature system is therefore unable to satisfy the metabolic needs of the developing cancer (15). This leads to areas within a tumor that have differing O_2_ tensions (anoxia, hypoxia, and normoxia) in combination with glucose deprivation, interstitial hypertension, and extra-cellular acidosis (16, 17). The differing compositions of TME between patients is thought to impact the effectiveness of different cancer therapies. If TME parameters such as biomarkers, cancer metabolites, chemotherapeutic drug concentrations or intra-tumoural pH and O_2_ could be analyzed over the course of a patient's treatment schedule, then this information could influence the management of the patient. Treatment could be tailored on the basis of the tumor's response to the initial therapy or through the identification of early markers of tumor recurrence or metastasis (18).

#### Tumor Hypoxia and pH

Low O_2_ levels, or hypoxia, is a regular feature of many tumors. Approximately 60% of solid cancers contain hypoxic areas heterogeneously dispersed throughout the tumor (15); this makes O_2_ a very relevant TME parameter to investigate. The vast majority of non-cancerous mammalian tissues operate in O_2_ levels of 2–9%. Hypoxic and severely hypoxic states, defined as ≤2% O_2_ and ≤0.02% O_2_, respectively (19), can occur within tumors via 4 main mechanisms:

1. Diffusion-limited hypoxia. Mainly leading to chronic hypoxia, diffusion-limited hypoxia is caused by viable aerobic cancer cells positioned close to blood vessels using the O_2_ transported in red blood cells (RBC). O_2_ levels decrease as the distance from the vasculature increases, resulting in hypoxia (20, 21). Even though O_2_ can diffuse up to 150–180 μm from a blood vessel, the distance at which hypoxia arises is dependent on the ability of RBC to carry O_2_, in addition to the metabolic needs of the cancer cells situated closest to the blood vessels (22).
2. Perfusion-limited hypoxia. This mostly leads to acute and transient hypoxia, and is caused by interruptions to blood flow (23, 24) resulting from structural/functional defects of micro-vessels within a cancer, which can lead to micro-vessel collapse/blockade (25). This may lead to complete ischemic hypoxia, or hypoxia that stops RBC flow but permits plasma flow to continue the provision of nutrients to the cancer cells.
3. Anemic hypoxia. Resulting from therapy-induced and/or tumor-associated anemia that causes decreased RBC O_2_ transport capabilities (20, 26).
4. Macroscopic regional hypoxia. Occurs due to the production of an O_2_ gradient over the length of a blood vessel. Blood present at the arterial extremity has the highest O_2_ levels, which is quickly consumed by tumor cells present in this area. O_2_ levels in the blood decrease as distance from the arterial end increases. As a result, cancer cells positioned at the distal end of the blood vessel may be hypoxic, even if they are close to the vessel (27, 28).

While it seems reasonable that low O_2_ levels would produce an adverse environment for cells to survive, tumor cells possess the ability to create a hypoxic response through alterations in gene expression that shield them from cell death mechanisms (29). Cancer cells that are incapable of adjusting to low O_2_ concentrations will perish, resulting in the selection of tumor cells with hypoxic-induced, genetically-fixed characteristics, known as a “hypoxic phenotype” (30). This hypoxia-driven malignant progression can lead to tumor cells becoming increasingly metastatic and therapy-resistant (31). There are a variety of crucial inducible transcription factors that participate in this hypoxic response, with hypoxia inducible factor-1 (HIF-1) being the one that has been most comprehensively studied so far (32–35). In areas where O_2_ levels are approximately 2% or less, these transcription factors are stabilized and transported to the cell nucleus, where they then bind hypoxia-response elements present in the promoter regions of target genes, leading to transcription (36). These hypoxia-regulated genes are involved in processes such as erythropoiesis, angiogenesis, apoptosis, autophagy, proliferation, glucose metabolism, intra-cellular acidosis, and metastasis. These adaptations enhance the capacity of cancer cells to endure these hostile low O_2_ TME regions (37).

In order to counteract the reduced mitochondrial ATP production that occurs due to low O_2_ levels, hypoxic cells can instead get their energy through anaerobic glycolysis, resulting in the generation of lactic acid, which can cause substantial acidosis within solid cancers (38). Even in normoxic conditions, some cancer cells still rely on glycolysis for energy production. This phenomenon, known as the Warburg effect, can lead to discrepancies in the spatial/temporal distribution of areas with low O_2_ concentrations and increased amounts of acidosis (39–41).

#### The Tumor Microenvironment and Its Effect on Treatment Responses

The heterogeneity that occurs within the TME can influence how tumors respond to frequently-employed cancer treatments and can subsequently have a negative impact on patient outcome. The abnormal cancer vasculature can inhibit the delivery of systemic drugs to the tumor, leading to heterogeneous drug dissemination within cancer tissue (42). In immunotherapy, high molecular weight drugs access cancer cells by the interstitial space rather than through blood vessels (43). Interstitial hypertension that can occur within tumors pushes fluid out of the interstitial space, thus restricting drug delivery (44, 45).

O_2_ levels within tissues can also affect tumor radiosensitivity and the efficacy of chemotherapeutic drugs (46). Radiotherapy works through damaging the DNA of cancer cells, either via the direct route (the ionizing radiation directly produces double or single strand breaks in the DNA) or the indirect route (ionizing radiation produces free radicals within the cancer cells, which subsequently cause DNA damage). It has been shown that tumor cells exposed to low O_2_ concentrations can tolerate radiation doses 2–3 times higher than that which can be withstood by normoxic cancer cells. The O_2_ fixation hypothesis has been suggested to explain the role of O_2_ in radiotherapy (19, 47). O_2_ present in cancer tissues reacts with DNA radicals produced as a result of radiation treatment, leading to the production of irreparable peroxy radicals. However, this reaction does not take place when O_2_ is not present; in these instances, damaged DNA strands can be repaired by free-radical scavengers such as endogenous thiols, granting these hypoxic cancer cells a considerable survival advantage (48). The effect that O_2_ has on radiotherapy was emphasized in a significant global clinical study which showed that pre-treatment O_2_ levels within head and neck tumors was a prognostic factor for survival post-radiotherapy (+/– chemotherapy, surgical intervention, or radiosensitizer) (49). While the form of hypoxia (i.e., acute or chronic) is unrelated to the initial radioprotective effect, cancer cells under chronic hypoxic conditions are usually more deprived of nutrients in comparison to acutely hypoxic cells. This nutrient deprivation could have a part to play in the cell's capacity to repair radiation-induced DNA damage; as such, acute hypoxic cancer cells may be more radioresistant than chronic hypoxic cells.

Reduced O_2_ levels and low pH conditions can also decrease the proliferation rates of tumor cells (50); this can in turn inhibit the efficacy of cytotoxic drugs that target rapidly-dividing cancer cells (51). Chemotherapeutic drugs such as doxorubicin (DOX) have also been shown to have increased efficacy in normoxic conditions; the intra-cellular metabolite of DOX reacts with O_2_ present in the TME, leading to the generation of reactive oxygen species (ROS). These ROS damage major components of the cell, leading to cellular death (52). This reaction does not take place in areas of the tumor where O_2_ levels are reduced (≤0.33%), leading to a reduction in the efficacy of DOX (53–55).

### Therapeutic Strategies to Target Hypoxic Tumors

Different treatment approaches have been assessed for their capacity to surmount hypoxia-related resistance to radiation. These strategies include hyperbaric O_2_ treatment, the administration of agents that target the hypoxic areas of cancers, or drugs that are activated in these low O_2_ regions. Hyperbaric O_2_ therapy, which uses high ambient air pressures to increase the amount of O_2_ carried in the patient's blood, has been found to have a beneficial effect in patients suffering from head and neck squamous cell carcinomas (HNSCC) (56); in spite of this, varying data produced from clinical trials in other cancer types, in addition to the logistical issues associated with its use, have hindered its general utility. Additionally, this form of therapy is not appropriate in combination with some chemotherapeutic drugs, including DOX, because it increases the likelihood of systemic ROS-mediated toxicities (57). A different method that has been used to increase O_2_ levels within tumors is the administration of carbogen with nicotinamide. Carbogen (also named Meduna's Mixture after its inventor) is a mixture of CO_2_ and O_2_ and has the ability to diminish diffusion-limited hypoxia, while nicotinamide is a vasoactive agent that counteracts acute hypoxia resulting from reduced perfusion. Accelerated radiotherapy in combination with carbogen and nicotinamide (ARCON) has been assessed in several clinical trials, demonstrating enhanced locoregional control and disease-free survival (58, 59). Likewise, clinical trials using nitroimidazole derivatives (e.g., nimorazole and doranidazole) that mimic the effects of O_2_, have shown that these drugs can produce survival benefits when combined with chemoradiotherapy in HNSCC (60) and non-small cell lung cancer (NSCLC) patients (61), or when used with radiotherapy alone (62, 63). In both Norway and Denmark, nimorazole combined with radiotherapy is the standard of care treatment for head and neck cancers (60, 62, 64). Additionally, the combination of radiotherapy with nicotinamide and carbogen has exhibited encouraging results in bladder and laryngeal cancers (65).

The identification of hypoxic tumor areas can also allow more effective delivery of radiation. Tumors that have large regions of hypoxia could be given increased radiation doses over the gross tumor volume; however, this method may lead to an escalated risk of damaging surrounding normal tissues. A superior technique would be to establish a biological target volume based on low O_2_ levels. With this approach the overall dose given to the gross tumor volume stays the same but it is redistributed to specific tumor regions; increasing the dose delivered to the low O_2_ areas, whilst decreasing that given to the regions with higher O_2_ concentrations. The biological target volume could also be given boost doses, either applied homogeneously over the hypoxic sub-volume, or altered in accordance with the local O_2_ levels. Improvements in radiation-delivery technology, including intensity-modulated and image-guided radiotherapy, enable highly conformal and accurate delivery of radiation; these developments mean that the use of sculpturing techniques or dose painting by numbers are becoming increasingly attainable (66). These new treatments are substantiated by modeling studies, which indicate that a boost dose of 10 Gy to low O_2_ regions in HNSCC patients could lead to a 17% increase in tumor control probability, without escalating the risk of complications (67). A different study has demonstrated that dose escalation, employing dose painting by numbers to the biological target volume, could also result in improved tumor control probability compared to that achieved with uniform dose escalation (68).

Case selection is a crucial feature of clinical trials designed to assess hypoxia-modifying therapies. Unfortunately, many previous studies enrolled patients into these clinical trials without initially determining the hypoxic nature of the tumors. Failure to specifically detect hypoxic tumors would inevitably lead to treating patients who were unlikely to gain any benefit from hypoxic modification. This issue leaves us with a crucial question: what is the best method to analyse O_2_ levels so clinicians can effectively decide which patients will benefit from therapeutic strategies designed to target hypoxic cancer cells?

### Tumor Functional Assessment

There are various methods by which hypoxic areas within tumors can be detected, but none are used routinely either due to their invasive nature or difficulties in incorporating them into clinical practice (Table 1). One of the first approaches for directly measuring intra-tumoral O_2_ was with the Eppendorf O_2_ electrode (70). This technique showed associations between hypoxia and treatment responses in numerous cancer types (71–73, 73–75) and that O_2_ concentrations within breast cancer tissue can be lower than that of normal breast tissue (70). However, this technique is invasive and only applicable to readily accessible tumors and is not used routinely in the clinic.

**Table 1:** Methods used for measuring tumor hypoxia.

**Microelectrode** **Technique:** O_2_ electrodes are put into solid tumors **Benefits:** Provides direct readings of O_2_ levels. Simple to use. Ability to acquire real-time readings **Drawbacks:** Invasive procedure. May only be utilized in tumors that are accessible. Incapable of differentiating viable hypoxic tumor regions from necrotic areas
**Tissue based biomarkers** **Technique:** Nitroimidazole compounds, such as pimonidazole, are given systemically and are changed into protein adducts in hypoxic cells. Detectable in biopsies through IHC **Benefits:** Provides an estimate of hypoxia heterogeneity in different cancer regions **Drawbacks:** Indirect measurement of O_2_ concentrations. Invasive procedure (biopsy required). May not distinguish intermediate hypoxic phenotypes (HIF-1 stabilization occurs at higher O_2_ levels than that at which adducts are formed). Real-time measurements are not possible
**Tissue based biomarkers** **Technique:** Assessing the expression of proteins induced in hypoxic conditions (GLUT1, CA9) **Benefits:** The location of protein expression and data on functional status can be provided through IHC. Gives an estimate of hypoxia heterogeneity within different cancer regions **Drawbacks:** Indirect measurement of O_2_ concentrations. Invasive procedure (biopsy required). Protein expression can be manipulated by factors other than hypoxia. Method limited to a small number of biomarkers due to antibody sensitivity and specificity issues. Real-time measurements are not possible
**Tissue based biomarkers** **Technique:** Assessing gene expression levels **Benefits:** Prognostic/predictive of response to hypoxic radiosensitizers **Drawbacks:** Indirect measurement of O_2_ concentrations. Invasive procedure (biopsy required). Gene expression can be manipulated by factors other than hypoxia. Real-time measurements are not possible
**Serological based biomarkers** **Technique:** Analysis of biomarkers present within the serum (osteopontin) **Benefits:** Non-invasive. Real-time measurements are possible **Drawbacks:** Indirect measurement of O_2_ concentrations. Biomarker levels can be manipulated by factors other than hypoxia. No information provided on hypoxic heterogeneity in different cancer regions.
**Positron emission tomography** **Technique:** Copper-complexed dithiosemicarbazone or nitroimidazole agents, given systemically, are taken up by hypoxic cancer cells. Agents are attached to radiotracers that can be distinguished through PET imaging **Benefits:** Non-invasive procedure. Capable of acquiring real-time data. Can provide an estimation of hypoxic heterogeneity in different cancer regions **Drawbacks:** Indirect measurement of O_2_ concentrations. May not distinguish intermediate hypoxic phenotypes (HIF-1 stabilization occurs at higher O_2_ levels than that at which agents form adducts). Safety issues with the use of radioisotopes
**Magnetic resonance imaging** **Technique:** Either blood oxygen level-dependent (BOLD) imaging, dynamic contrast-enhanced MRI (DCE-MRI) or oxygen-enhanced MRI (OE-MRI) **Benefits:** Non-invasive procedure. Real-time measurements can be obtained. Can provide an estimation of hypoxic heterogeneity in different cancer regions. Radiation exposure avoided **Drawbacks:** Possibility of artifacts. The parameters used to reflect O_2_ levels and their link with hypoxia is yet to be determined

*Advantages and disadvantages of each method used to measure tumor hypoxia (69)*.

While the Eppendorf O_2_ electrode represents a direct method of measuring O_2_ concentrations within cancer tissues, there are also various indirect methods that can be utilized. The use of cancer tissue biopsies to identify molecular reporters of O_2_ is one such indirect method. Nitroimidazole-based agents, such as pimonidazole, are chemicals that produce adducts with intra-cellular macromolecules in reduced O_2_ concentrations (76); pimonidazole has been utilized successfully in clinical trials to choose patients that would be suited for treatment with hypoxia-modifying drugs alongside accelerated radiotherapy (58, 59). To exploit the cellular response of cancer cells to low O_2_ levels, the expression levels of hypoxia-induced genes, mRNAs, and proteins have also been employed as biomarkers of hypoxia (77–80). In dogs with mammary tumors, high *VEGFA* gene expression has been associated with poor outcome, with one study suggesting that it could be used as a prognostic marker to identify dogs at risk of disease progression (81). However, markers including HIF-1, CA9, VEGF and GLUT1, analyzed either at the mRNA or protein level, or through the use of nitroimidazole, have frequently brought about contradictory results. This is probably because these genes and proteins can be regulated by factors other than O_2_, such as glucose levels or extracellular pH (82–84), or their expression is induced at O_2_ concentrations not low enough to have a significant radiobiological effect. For nitroimidazole to bind to macromolecules it requires cells to be hypoxic for long periods of time. Therefore, this can result in an underestimation of the levels of acute hypoxia within the TME.

These problems led to the production of hypoxic signatures that were created by pinpointing a variety of genes that were upregulated in low O_2_ conditions; these signatures were produced from either cell lines or clinical tissues (85–92). The Toustrup15-gene-classifier is one such signature that was generated from a panel of genes ascertained using HNSCC cell lines; these genes were found to be upregulated by hypoxia, independent of pH (85). This gene signature was further developed in a training cohort of 58 patients suffering from HNSCC, with O_2_ levels analyzed with an electrode. The classifier was validated in a Danish study where patients were randomly selected to be treated with radiotherapy combined with either the hypoxic radiosensitizer nimorazole or a placebo. The classifier was shown to be prognostic and also possessed predictive power for hypoxic modification (85, 93, 94). The 26-gene signature generated by Eustace et al. (91) is another classifier based on a metagene signature generated from patients suffering from lung, breast and head and neck cancers. The ARCON trial carried out in the Netherlands assessed this signature, evaluating radiotherapy combined with nicotinamide and carbogen compared to radiotherapy alone in patients with laryngeal cancer. The patients that were classed as “more hypoxic” with the 26-gene signature exhibited significantly better locoregional control when treated with hypoxia-modifying compounds (44, 59).

Indirect assessments of tumor hypoxia rely on the ability to obtain a tissue sample. Unfortunately, these can be extremely challenging or even impossible to acquire due to tumor location or the patient's condition. Treatment response monitoring through repeat biopsies is also difficult to clinically justify. The measuring of hypoxic-related biomarkers in blood samples has been investigated as a means of overcoming these issues. In humans, high levels of plasma osteopontin have been associated with a poor prognosis in head and neck cancers, which could be improved with O_2_-mimicking drugs (95). Serum concentrations of HIF-1α and VEGF in dogs with mammary tumors have also been shown to have prognostic merit; high VEGF levels were associated with lymph node and distant metastasis, tumor vascularization and decreased survival times, whereas high HIF-1α levels were related to local recurrences or metastatic lesions (81). The major disadvantage of using blood to evaluate the hypoxic status of tumors is that the sample, in effect, does not analyse the tumor at all. This means that specific hypoxic tumor areas cannot be identified and alterations to the O_2_ distribution within the tumor cannot be assessed. Tumor hypoxia assessment through non-invasive advanced imaging techniques, such as positron emission tomography (PET) and magnetic resonance imaging (MRI), offer the ability to evaluate the whole tumor volume whilst providing a means for repeat measurement.

Dynamic contrast-enhanced MRI (DCE-MRI) typically uses an intravenous injection of a gadolinium-based contrast agent. Signal changes identified by MRI as the agent passes through the tumor's blood supply provide an indication of intra-tumoral perfusion. Blood O_2_ level-dependent (BOLD) MRI imaging detects signal alterations caused by changes in deoxygenated hemoglobin levels. Patients are required to undergo dynamic challenges to alter deoxygenated and oxygenated hemoglobin ratios in order to map hypoxic tumor regions (69). Over the years, PET imaging has been developed to assess specific tumor characteristic such as glycolysis (2-deoxy-2-[^18^F]fluoro-D-glucose [^18^F-FDG]), hypoxia ([^18^F]fluoromisonidazole [FMISO], CA9, copper-complexed dithiosemicarbazone [^64^Cu-ATSM]), and proliferation (3′-deoxy-3′-[^18^F]fluorothymidine [^18^F-FLT]). ^18^F-FDG, ^64^Cu-ATSM, and ^18^F-FLT have all shown promising results for monitoring treatment responses in numerous human and canine cancer types (96–100).

One canine study demonstrated the feasibility of using multiple PET tracers to simultaneously evaluate glycolysis (^18^F-FDG), hypoxia (^64^Cu-ATSM), and proliferation (^18^F-FLT) in a dog diagnosed with a tibial fibrosarcoma. Serial imaging performed before, during and after 10 fractions of 4.5 Gy demonstrated a heterogeneous spatial distribution of the 3 tracers. ^64^Cu-ATSM uptake progressively decreased during and after radiotherapy, suggesting either tumor reoxygenation (in areas where ^18^F-FDG uptake was maintained) or the development of dead tumor tissue (in areas of reduced ^18^F-FDG uptake). Although ^18^F-FDG and ^18^F-FLT uptake significantly decreased following the completion of radiotherapy, fluctuations were seen throughout the treatment course. ^18^F-FLT fluctuations could be indicative of accelerated tumor cell repopulation, whereas ^18^F-FDG fluctuations might suggest the presence of inflammation or tissue remodeling. The authors put forward the suggestion that each tracer provided distinct information about the TME, including heterogeneity, phenotype and response to treatment, which could be used to tailor therapies to individual patients (101). Other canine studies have used ^64^Cu-ATSM and ^18^F-FDG to identify biological target volumes for dose painting radiotherapy (102), with ^18^F-FLT and ^18^F-FDG also having been investigated as a means of monitoring radiotherapy responses and identifying disease progression (103). Pre-treatment ^18^F-FDG, ^18^F-FLT, and ^61^Cu-ATSM scans could also be used to predict the response to radiotherapy (104).

Unfortunately, these advanced imaging techniques are not without limitations; in particular, the resolution (i.e., voxel size; a 3D representation of a pixel as determined by slice thickness and pixel size) offered by these techniques can be greater than the hypoxic tumor areas themselves. This means that small hypoxic “hotspots” may be classified as normoxic if the mean value for the area falls below a specific cut-off point. In clinical terms, PET imaging may not be able to accurately characterize intra-tumoral hypoxic heterogeneity at microregional levels.

Currently, regardless of whether tumor hypoxia, glycolysis, or proliferation are assessed using tissue or blood samples or through advanced imaging, the results only provide a static indication of the TME at the time of analysis. This is a significant limitation of any technique, as the TME is a dynamic landscape with potentially significant spatial and temporal changes occurring throughout the tumor at any given time. Therefore, despite technological advances, clinical techniques that can acquire continuous real-time data in order to generate an accurate 3D map of the TME, including its hypoxic status, within the entire tumor volume are still not available. A potential solution to this unmet clinical need is the use of an implantable sensor designed to continually measure key TME parameters. This technique has the potential to provide information on the spatial and temporal changes that occur within the tumor during a patient's treatment course.

### Implantable Technology and Cancer

**Figure 2:**
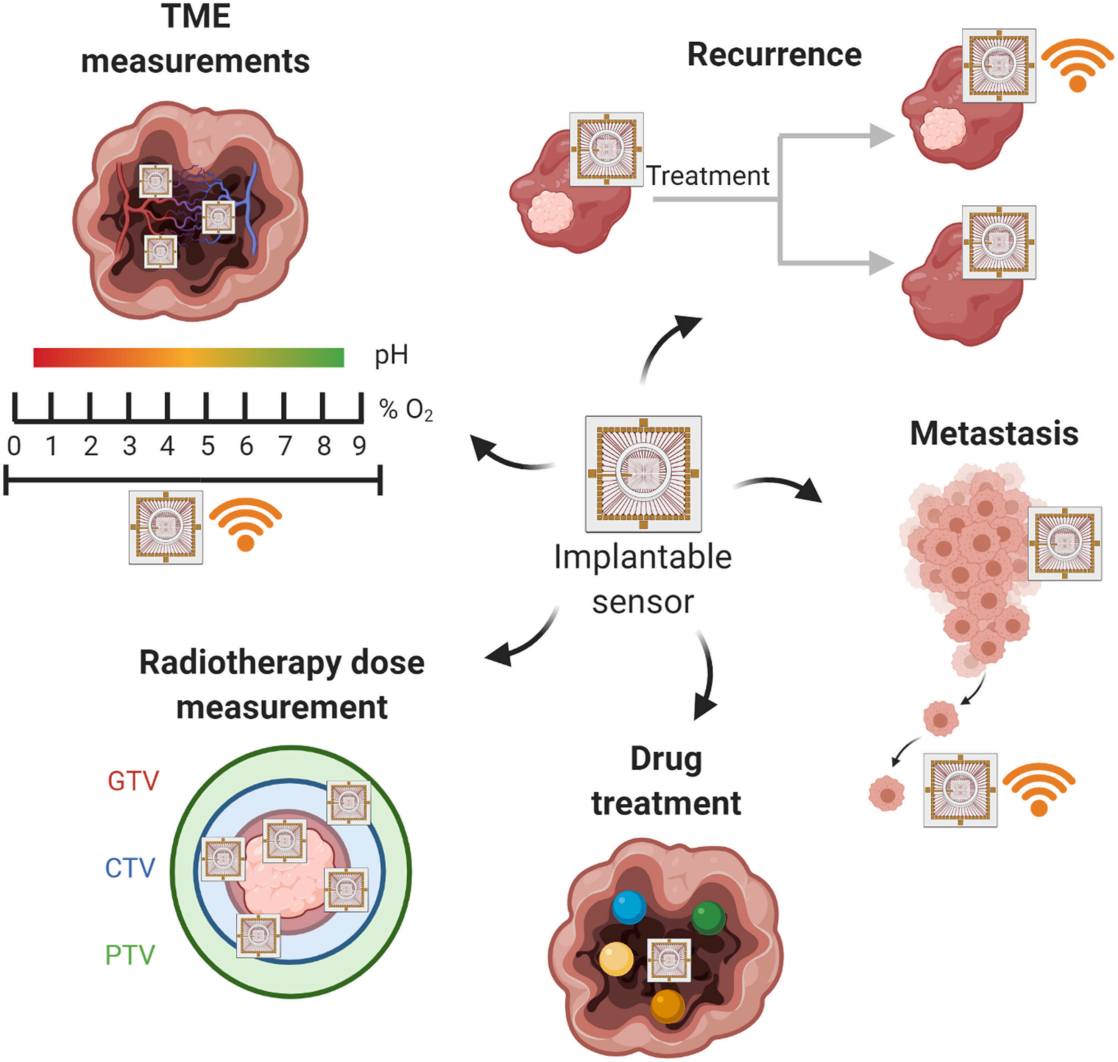
Applications of implantable technologies for precision medicine. GTV, gross tumor volume; CTV, clinical tumor volume; PTV, planning target volume. Figure created with BioRender.com.

Developments in the fields of electronics, mechanical engineering, and microfabrication have resulted in increased interest in the clinical applications of implantable medical devices for precision medicine. In line with this, studies have begun investigating whether implantable devices could be used to map the TME for parameters such as O_2_, deliver local drug treatments, assess treatment responses, and monitor for tumor recurrence and metastasis (Figure 2).

#### Implantable Sensors and Monitoring of Tumor Oxygenation and pH

Due to the limitations associated with current technologies designed for measuring intra-tumoral O_2_ levels, researchers are investigating novel ways to address this unmet clinical need. Two recent studies have developed implantable sensors that are able to provide O_2_ and pH measurements within a tumor.

The Implantable Microsystems for Personalized Anti-Cancer Therapy (IMPACT) project is developing both miniaturized Clark-type electrochemical O_2_ sensors, methylene blue-based electrochemical pH sensors and ISFET pH sensors (105). The purpose of the project is to manufacture implantable wireless sensors for the real-time monitoring of intra-tumoral O_2_ levels and pH, thus allowing radiotherapy to be delivered at the most effective location and time. *In vivo* mouse xenograft tumor studies using a human breast cancer cell line were used to evaluate biofouling and the foreign body response that occurred when constituent materials of the IMPACT sensors were implanted into a solid tumor. This study was the first to assess the effects of modern implantable materials within a TME. The authors concluded that none of the evaluated materials had any detrimental effect on tumor growth or body weight of the murine host. Up to 14 days post-implantation, immunohistochemistry (IHC) showed no significant changes in hypoxic cell number, tumor necrosis, apoptosis, proliferation, collagen deposition, or immune cell infiltration. The authors suggested that the absence of biofouling supports the use of these materials in medical devices designed for implantation within solid tumors (106). Functional O_2_ sensors were subsequently evaluated in a translational large animal lung cancer model (107). Following CT-guided implantation into lung tumors, initial results have demonstrated that these sensors remained functional and were sufficiently sensitive to monitor acute changes in oxygenation within tumor tissue (108).

Other researchers have taken a different approach to monitoring TME parameters. One such study has designed a miniaturized implantable nuclear magnetic resonance (NMR) sensor. This incorporates a responsive NMR contrast agent that can be assessed wirelessly via magnetic coupling to an external reader without the need of an MRI scanner. This study demonstrated the feasibility of the implantable NMR sensor to separately detect pH and O_2_ using *in vitro* and *in vivo* model systems. The *in vivo* experiments consisted of monitoring intra-tumoral pH in a xenograft mouse model and assessing O_2_ levels in a rat hind limb constriction model. The authors suggested that this sensor could be implanted at the time of tumor biopsy and could remain within the tumor providing repeat O_2_ and pH measurements from the same tumor area. They further suggested that the sensor could be used for other NMR applications, such as low-resolution spectroscopy to identify soluble biomarkers (109).

Although further engineering development of both sensor types is required, the results from these exciting pre-clinical studies have shown that intra-tumoral O_2_ and pH can be monitored in real-time. If they progress toward testing in clinical trials, they could eventually prove to be formidable tools to identify and target treatment-resistant tumor areas.

#### Implantable Sensors and the Clinical Monitoring of Cancer Progression

The ability to monitor treatment responses and identify early indicators of tumor recurrence may be achieved through the use of implantable medical devices. Sensors that detect cancer-related biomarkers could be implanted either within the tumor at the time of biopsy or within the tumor bed at the time of surgery following tumor excision (110, 111). This application would be highly useful in disease conditions where advanced imaging cannot reliably distinguish between necrosis/fibrosis resulting from previous surgery/chemotherapy/radiotherapy and tumor recurrence (e.g., glioblastomas) (112).

The first *in vivo* report to support the use of implantable devices for this application was designed to detect the β subunit of human chorionic gonadotrophin (hcG-β) (111), a soluble cancer-related biomarker known to be secreted in high levels in ovarian and testicular cancers. The sensor was designed using nanoparticle magnetic relaxation switches (MRSw) conjugated with hcG-β antibodies and was evaluated using a mouse xenograft model. Sensors were implanted into tissue surrounding hcG-β-producing human choriocarcinoma xenograft tumors (JEG-3). The results demonstrated that MRI was able to identify MRSw aggregation that occurred with hcG-β antibody binding, which provided evidence that the implanted device could successfully detect hcG-β in peri-tumoral tissue. The authors suggested that, as MRSw can be modified to detect various molecules, they might provide a means of detecting and monitoring a variety of cancer-related biomarkers (113).

Another study has investigated the use of implantable devices designed to identify early indicators of metastasis through the evaluation of localized immune responses. This study developed a synthetic microporous polymer scaffold that promotes tissue ingrowth through vascularization, cellular infiltration, and immune cell recruitment. This engineered microenvironment was shown to have characteristics of a metastatic niche that could capture circulating tumor cells. *In vivo* evaluation of the immune responses that developed within subcutaneously-implanted scaffolds was performed using an orthotopic breast cancer murine xenograft model. Phenotypes and gene expression profiles of immune cells were determined from sequential scaffold biopsies obtained up to 21 days post-primary tumor inoculation. These results suggested that an immunosuppressive microenvironment developed which had characteristics of metastatic diseased lung. Following excision of the orthotopic breast tumor, sequential analysis of immune phenotypes within the scaffold showed an initial response to surgery, which could differentiate mice that suffered a tumor recurrence from those that survived. The authors concluded that the microenvironment that developed within the scaffold reflected the immunosuppressive events that contribute to the development of metastatic disease. Furthermore, monitoring these immune responses identified animals which had disease progression from those that responded to surgery (114). Technologies such as this may provide a means of identifying patients at risk of developing metastatic disease or those who have early metastatic disease that is clinically undetectable by imaging. Patient outcomes have the potential to be improved if the earliest stages of metastatic disease can be identified and treatment intensification initiated.

Implantable devices can not only be designed to monitor specific tumor-related factors but may be utilized to assess the accuracy of delivering a desired radiation dose to a specific tissue/tumor area, an issue which is fundamental to the clinical effectiveness and success of radiotherapy. To address this, implantable dosimeters have been developed to assess what radiation dose has been delivered per treatment fraction to the planned target volume. This dose-verification system has gained FDA approval for use in prostate and breast cancers, and has the potential to allow radiotherapy treatment regimens to become optimized for individual patients (115).

#### Cancer Treatment and Implantable Technology

Although many cancer types can be treated with a variety of clinically-approved drugs, clinicians are often faced with the difficult decision of which drug would be most effective in individual patients. Sometimes this decision can be made based on published evidence, but occasionally the choice will be empirical and based solely upon the clinician's personal experiences. To overcome this issue, studies have investigated whether multiple drugs can be simultaneously evaluated in the tumor itself to provide information on which is most effective. These types of studies have the ability to account for tumor heterogeneity, as drug effects can be analyzed within spatially distinct TME regions.

One study developed a medical device that was able to deliver 8 drugs concurrently into mapped and defined regions within a tumor. This research initially used lymphoma and NSCLC murine xenograft models for validation of the device. Following intra-tumoral injection, drug distribution was assessed through radiolabelling imaging. Tumors were excised up to 72 h after drug delivery and cytotoxic drug responses were evaluated through IHC. Results demonstrated that not only could local tumor responses predict response to systemically-delivered drugs, but that the most effective compound for a chemoresistant lymphoma xenograft tumor could be identified by screening multiple drugs simultaneously. This work was followed by a small clinical study designed to assess patient satisfaction and procedural complications. Using the device, combined with ultrasound guidance, human and canine lymphoma patients received microinjections of vincristine into enlarged lymph nodes. Results suggested that localized tumor responses can be tested in a toxicity-sparing manner (116). Employing a similar approach, a further study developed a short-term implantable device composed of multiple reservoirs. Following intra-tumoral implantation, each reservoir was capable of delivering drugs into spatially distinct TME areas at concentrations equivalent to systemically-achievable doses (117). 24 h after implantation, the device and the immediately adjacent surrounding tumor tissue was removed with IHC performed to assess each drugs' cytotoxic effect (117).

These studies demonstrate that implantable devices can be used to conduct *in vivo* drug testing through local delivery directly within the TME. The ability to evaluate spatially-defined tumor responses to multiple drugs provides an opportunity to identify a patient's optimal drug treatment before definitive systemic chemotherapy begins.

A further consideration for determining the most appropriate chemotherapy regimen for a patient is the issue of multidrug resistance (MDR). MDR occurs when cancer cells develop cross-resistance to multiple functionally and structurally unrelated chemotherapeutic agents. One of the most common MDR mechanisms seen in various cancer types is related to the expression of phosphoglycoprotein multidrug resistance protein 1 (MRP1), part of the ATP-binding cassette. High MRP1 expression has been correlated with reduced tumor responses to many chemotherapeutic agents, including 5-fluorouracil (5-FU), along with reduced overall survival times. In order to address this issue, a study has developed a bio-responsive hydrogel-nanoprobe comprised of a 5-FU-intercalated DNA hairpin. This nanoprobe was designed to locally detect and bind to a complementary *MRP1* target sequence within the TME, resulting in *MRP1* silencing and inhibition of protein expression. Binding of the DNA hairpin to the *MRP1* sequence also caused a conformational change to the nanoprobe, which resulted in release of 5-FU. A 5-FU resistant breast cancer murine xenograft model was used to evaluate the ability of these nanoprobes to detect and overcome MDR. *In vivo* imaging was performed for 14 days following the implantation of a hydrogel disc loaded with nanoparticles into a xenografted tumor. Luciferase expression was utilized to assess tumor inhibition, while FITC fluorescence emission could identify nanoprobes before and after hybridization to MRP1 mRNA. Results indicated that, despite 5-FU resistance, more than 90% tumor reduction was achieved following 80% MRP1 silencing. The authors suggested that this approach could not only be used to reverse 5-FU resistance, but that it could also be used to reverse resistance to other chemotherapeutic agents and improve treatment responses (118).

As opposed to targeting specific resistance mechanisms, other studies have investigated ways in which the cytotoxicity of chemotherapeutic agents can be enhanced. A novel solution to this is through the use of implantable O_2_-generating depots; these are designed to improve DOX cytotoxicity by promoting ROS production through their ability to increase intra-tumoral O_2_ concentrations, without affecting systemic levels. Calcium peroxide (CaO_2_) alginate microencapsulated pellets have been developed to react with interstitial fluid, forming calcium hydroxide [Ca(OH)_2_] and hydrogen peroxide (H_2_O_2_); it is this H_2_O_2_ which then decomposes to release O_2_. These pellets have been shown to successfully reduce the hypoxic TME of Hep3B xenograft tumors and increase the chemotherapeutic effect of DOX following their implantation into peri-tumoral tissue. The results from this study demonstrated the feasibility of using this O_2_-generating system to locally enhance the cytotoxic effects of DOX and overcome hypoxia-induced DOX resistance (53).

### Precision Medicine in Veterinary Clinics

Although veterinary precision medicine has yet to be incorporated into routine clinical practices, there are encouraging results from pre-clinical and clinical research studies that provide examples of how it could be used in the treatment of veterinary patients.

The development of the first canine-specific anti-cancer drug, toceranib (Palladia™), is the current closest example of veterinary precision medicine routinely used in the clinics. Toceranib is a novel multi-receptor tyrosine kinase inhibitor (119). Following pre-clinical studies demonstrating its anti-proliferative (*in vitro* cell line models) and anti-angiogenic activity (*in vivo* murine xenograft models), phase I trials were undertaken (120). These trials were conducted in dogs diagnosed with various tumor types; all patients had a guarded prognosis as they had either failed standard treatment regimens or there was no available therapeutic alternative. Results from this trial and subsequent studies showed toceranib had the greatest tumor response rates in mast cell tumors (MCT) (121). Toceranib subsequently gained clinical approval for the treatment of grade II and III cutaneous MCT. Although this drug was not developed as part of a precision medicine approach, further investigation of its mode of action demonstrated that MCT with a specific *c-KIT* mutation had better response rates compared to those that had no mutation (60 vs. 31% response). This *c-KIT* mutation was identified as an internal tandem duplication that alters *KIT* expression and causes constitutive receptor phosphorylation. Unlike clinically-approved kinase inhibitors in human medicine, the approval granted for toceranib did not stipulate its use solely for MCT with specific *c-KIT* mutations largely because some dogs without mutations still responded to treatment. The situation is also complicated by the issue that there is no clinically-approved canine genetic test for *c-KIT* mutations. Therefore, while toceranib is the closest, evidence-based, precision medicine approach in veterinary medicine, it currently fails to meet important considerations in which human-targeted therapies are based (122).

The use of circulating tumor cells (CTC) and transcriptomics to provide precision veterinary medicine through the identification of prognostic, predictive or treatment response information has also been investigated in multiple studies. CTC from canine mammary tumors have been shown to correlate with metastatic disease development and provide prognostic information that can be used to determine more aggressive treatment regimens for high-risk patients (123). Similar approaches have used transcriptomics to generate prognostic mRNA signatures from canine mammary tumors and lymphomas. These results demonstrated that the transcriptome could indicate malignancy and metastatic potential, which again could be used to identify high-risk patients requiring treatment intensification (124–127). Canine lymphoma mRNA expression signatures have also been associated with grade, immuno-phenotype and therapeutic response. The authors of this study successfully developed a real-time PCR-based test which they suggested could be easily adopted for use in clinical cases (128). A further exciting canine lymphoma study investigated the assessment of minimal residual disease and response to treatment (129). To monitor the response to chemotherapy, this study used a real-time PCR assay to detect immunoglobulin heavy chain gene fragment sequences in the blood of dogs diagnosed with high-grade B-cell lymphoma. Results showed that the assay could predict the 25 week progression-free survival from as early as the 11th week of treatment. Translated into the clinics, these results would enable clinicians to identify non-responding patients early in their treatment; these patients could either be transferred onto a different treatment protocol or, at the very least, be spared the side effects and costs of continuing an ineffective chemotherapy regimen.

Another study has recently reported the use of a cross-species personalized medicine approach to identify new therapies for a dog diagnosed with multiple leiomyosarcomas. This study used tissue obtained from an excisional biopsy from 1 of the tumors to establish patient-derived xenograft (PDX) tumors. Using PDX samples, an *in vitro* cell line was subsequently established that was used for high-throughput drug screening. This *in vitro* work identified proteasome inhibitors as a potential therapy, which was then validated using the PDX model. Genomic profiling of mutations in the original tumors, PDX tumors and cell line was also performed. While these investigations were taking place, treatment with toceranib began. After 6 months, disease progression and local recurrence were detected; the dog was then treated with the proteasomal inhibitor bortezomib. Although an initial response was seen, tumor growth began again 6 weeks after the start of treatment and the dog was euthanized. While this study demonstrated that drug screening can be performed on patient-derived samples, the time scale that this occurred over was approximately 1 year, by which time that patient had developed metastatic disease and local recurrence. The major issue with this study is that these metastatic lesions may not have had the same oncogenic drivers as the original tumor, and therefore the use of bortezomib may not have been appropriate. This dog also had 6 original tumors, but cell lines and PDX tumors were only made from 1; tumor heterogeneity between the tumors should also have been evaluated. Although the study demonstrated what can be achieved through using patient derived samples, the technique is impractical in terms of expense and the time required to complete the work. These types of studies can only really be effective if treatment strategies can be individualized for the patient in order to treat the primary disease at the time of diagnosis (130).

In contrast to the traditional forms of cancer therapy such as surgery, radiotherapy and chemotherapy, research has shown the potential benefits of using immunotherapy in a variety of cancer types. Studies have investigated whether personalized immunotherapy can be achieved through the production of autologous therapeutic anti-tumor vaccines using hydroxyapatite (HA). HA is an appealing compound for this application, as it not only attracts antigen-presenting cells, but also presents tumor antigens to immune cells. Studies have shown that cell membrane and heat shock proteins extracted from tumor tissue can be combined with HA to produce a personalized vaccine. Using this approach, a clinical trial demonstrated that 15% of patients gained a partial response, while 25% obtained stable disease following vaccination. The authors suggested that the vaccine stimulated a T-cell response and that personalized vaccines using HA combined with self-antigens were safe to use in patients (131). This approach has subsequently been investigated in dogs diagnosed with diffuse large B-cell lymphomas (DLBCL) (132). Results from this clinical trial demonstrated that dogs which received chemo-immunotherapy had longer progression-free and lymphoma-specific survival times compared with dogs that received only chemotherapy. These results suggested that the personalized vaccine was safe in dogs and could be used to improve treatment outcomes in dogs with DLBCL. The authors proposed that this novel therapeutic strategy should be investigated in human patients diagnosed with DLBCL.

Other studies have shown that immunotherapies using chimeric antigen receptor (CAR) T cells may have value in treating human patients with B cell neoplasia; the FDA approved the use of CD19 CAR T cells for treating refractory/relapsed acute lymphoblastic leukemia and DLBCL (133). In line with these human studies, a recent first-in-species pilot trial investigated the use of CAR T cells in canine DLBCL patients. Using blood samples obtained from dogs diagnosed with DLBCL, CD20 CAR T cells were produced *ex vivo* using lentivectors, as had been previously done to manufacture human CAR T cells. Results showed that canine CAR T cells could be detected in blood samples post-infusion, and that these cells were antigen-specific, resulting in removal of CD20^+^ target cells. Survival times also correlated with *ex vivo* CD20 CAR T cell expansion. Unfortunately, the induction of canine anti-mouse antibodies in the dogs resulted in CAR T cell loss. Furthermore, targeting CD20^+^ cells eventually resulted in antigen escape and the emergence of CD20^−^ disease. However, the study was able to show the successful lentivector production of functional canine CAR T cells, while also demonstrating that the challenges of effective CAR T cell therapy in animals were comparable to those seen in humans trials (134).

Osteosarcoma is another human and canine tumor type that may benefit from immunotherapies. Results from both canine (135–137) and human (138, 139) studies indicate that osteosarcoma patients which develop a surgical infection following limb sparing surgery have improved survival times. Although the immune mechanisms that lead to higher survival rates after infection are not yet clear these results suggest an immunogenic component to osteosarcoma pathogenesis. Studies using a mouse model of chronic bacterial osteomyelitis suggest that the innate immune system may be involved in the suppression of osteosarcoma growth (140). Immune modulation has produced positive effects on patient outcome in both dogs and humans; further studies are currently underway to ascertain the most effective immunotherapy or combination of immunotherapies that will lead to additional improvements in clinical response. With a better understanding of the methods required to redirect the immune system toward osteosarcoma, there is now ample opportunity to change the therapeutic landscape and improve osteosarcoma treatment in both humans and dogs (141, 142).

Other veterinary research projects have characterized the genotypes of several canine cancers including hemangiosarcoma (143), melanoma (144), and osteosarcoma (145), with the aim of identifying potential “actionable” targets. Although somatic mutations in these cancer types (in genes including *TP53, PIK3CA, NRAS, PTEN*, and *BRAF*) have similarities to those oncogenic mutations found in human cancers, veterinary clinical trials still need to be performed to confirm if they respond to targeted inhibition. Clinical trials that merge information gained from sequencing individual canine tumors with survival data after targeted treatment could in the future influence the selection of treatment regimens for canine cancer patients. These types of trials have the potential to provide evidence to clinicians and clients that cancer genome sequencing for their pets could lead to improved outcomes though the adoption of a precision medicine approach.

### Current Issues Limiting the Use of Precision Medicine

The translation of precision medicine approaches into accepted healthcare practices and policies has undoubtedly lagged behind the significant discoveries made through pre-clinical research. It is to be expected that, as with all novel innovations, it will take years to achieve this successful translation. Precision medicine is complicated by the fact that it not only represents a significant paradigm shift in how we treat cancer patients, but is also associated with complex legal, financial, social and ethical issues. The National Academy of Medicine outlined multiple challenges faced by the Precision Medicine Initiative, including the development of infrastructures that enable data sharing and easy access to extensive, highly-integrated clinical data sets. They also highlighted the absolute need for evidence that precision medicine can actually improve patient outcomes (146). To date, only a few clinical trials have assessed the adoption of a precision medicine approach for the treatment of cancer patients. The first of such precision medicine clinical trials involved refractory metastatic cancer patients. The results demonstrated that longer progression-free intervals could be achieved in patients that underwent molecular cancer profiling to guide their treatment (147). Unfortunately, other studies have suggested that there is limited evidence to support the use of genomic tests in healthcare practices (148, 149). The economic feasibility of adopting precision medicine policies is another significant challenge, as its successful implementation will ultimately depend on whether patients and healthcare providers are willing to pay for them. Currently, there is limited evidence that precision medicine provides sufficient cost-benefit advantages over standard treatment protocols. Some researchers even suggest that precision medicine could be a distraction from low-cost and effective population-wide interventions and that policies which focus on public health and prevention should be prioritized (150). Finally, precision medicine raises important questions regarding patient trust, including issues related to who actually owns the genetic data and how it can be securely stored.

To address some of these issues, centers such as The Personalized Medicine Coalition in the USA and The Center for Personalized Medicine in the UK have been established (151). Other on-going initiatives include the Clinical Sequencing Evidence-Generating Research center and the Implementing GeNomics In practice (IGNITE) project, which aim to support the integration of genome sequencing into healthcare practices (8). In veterinary medicine, following sequencing of the canine genome, the Canine Comparative Oncology and Genomics Consortium (CCOGC) was formed in 2004. This programme aims to generate an extensive archive (from 3,000 dogs) of clinical samples (neoplastic and non-neoplastic tissue, whole blood, plasma, serum and urine) from lymphoma, osteosarcoma, hemangiosarcoma, mast cell tumor, pulmonary, melanoma and soft tissue sarcomas. In 2013, the CCOGC biorepository was opened to the research community with the aim of improving the molecular characterization of canine cancers in order to help provide precision veterinary healthcare (152).

The challenges that limit the successful integration of precision medicine in human healthcare practices are equally applicable to veterinary medicine. As public awareness of the potential human benefits of precision medicine increases, veterinary clients will start to ask whether similar, precision-guided approaches can benefit their pets. This will become even more evident if pet owners themselves have received treatment as part of a precision medicine approach. In the future, the veterinary profession will need to adapt to the changes that are associated with precision medicine. Significant investment will be required to encourage openness for sharing research results and clinical data. Clinicians will also need to be trained in the use of molecular-based diagnostic assays and in performing high quality clinical trials using appropriately identified targeted treatments. Underpinning these ideas will ultimately require training and educational programmes for veterinary clinicians and veterinary healthcare professionals in subject areas such as tumor heterogeneity, genomic medicine, bioinformatics and targeted therapeutics, all of which are key components within human precision medicine.

## Conclusion

Precision medicine has the potential to transform the way in which we treat human and veterinary cancer patients. Ongoing basic and clinical research is continuing to improve our understanding of tumor heterogeneity and is providing new information on how it influences disease prognosis and treatment responses. The challenge lies in the successful translation of these exciting results into novel diagnostic and therapeutic strategies for clinical use. Multidisciplinary projects that take on a one-health view to precision medicine, involving human and veterinary clinicians, engineers and chemists, are likely to become more important in the future if we are to ultimately advance the field of precision medicine to a stage where it can be fully integrated into clinical practices. Our review has highlighted this with studies investigating the TME and with research using novel implantable medical devices. Although precision medicine is still largely focused on human healthcare, we have shown that there have been exciting clinical research studies in the field of veterinary precision medicine, with promising results. These types of studies are likely to increase as more researchers realize that many of the cancers seen in veterinary patients share significant similarities with human equivalent cancers, including the molecular mechanisms of disease pathogenesis and the TME. It is these types of similarities that may prove to be clinically relevant and therapeutically actionable for both human and veterinary patients. Adopting a one-health approach, in which researchers and clinicians from both human and veterinary fields collaborate, will ultimately aid translational studies and improve the integration of precision medicine initiatives into healthcare practices for both human and veterinary cancer patients.

## Author Contributions

DA secured funding for this research. MG wrote the majority of the manuscript and composed the figures, with significant contributions from JM. Critical revisions were made by MG, JM, AT, CM-P, CK, LP and DA. All authors read and approved the final manuscript.

## Conflict of Interest

The authors declare that the research was conducted in the absence of any commercial or financial relationships that could be construed as a potential conflict of interest.
